# Living Lab: Design a digital health intervention for healthy diet of ethnic minority adolescents

**DOI:** 10.1093/eurpub/ckac131.363

**Published:** 2022-10-25

**Authors:** J Lee, H Lee, H Lee, K Konlan, J Seo

**Affiliations:** 1 Brain Korea 21 FOUR Project, Yonsei University, Seoul, South Korea; 2 Mo-Im Kim Nursing Research Institute, Yonsei University, Seoul, South Korea; 3 College of Nursing, Yonsei University, Seoul, South Korea

## Abstract

Eating habits cultivated during adolescence continue through adulthood, and can widen the health gap in adulthood for vulnerable ethnic minority adolescents (EMA). Living Lab is a methodological approach through which stakeholders co-create innovations as citizen scientists in real-life settings. This study aimed to design a digital health intervention (DHI) for enhancing the heathy diet of EMA using the Living Lab approach. The DHI’s content and strategies were derived through literature reviews and focus group interviews. The Living Lab was structured by using 5 principles: real-life setting, user engagement, multi-stakeholder participation, multi-method, and co-creation. It has four activity phases of discovering problems, exploring solutions, solving problems, and disseminating solutions putting more emphasis on the use of digital device and multiple stakeholders such as peers and teachers in co-ideation. DHI participants were grouped into equal proportions of EMA and Korean-ethnic peers. The DHI operates for 2 hours once a week for 4 weeks, with orientation and wrap-up sessions before and after the DHI. Each activity of the co-working process is designed by applying behavior change techniques such as prompts/cues, framing/reframing, and credible sources in a digital educational environment: creating content using Google Jamboard and Padlet, and working on the metaverse platform ZEP. The responses and feedback from the participants are received through an online reflection diary weekly. Usability and acceptability of digital technology are assessed by an online survey on completion of the DHI. The efficacy of DHI is assessed through the change in dietary behavior and food literacy. This study was designed to enable EMA recognize the harmful effects of an unhealthy diet and co-create solutions through dynamic activities in a digital environment. Further, it may serve to change the cultural sensitivity of native peers that influence the health choices of EMA.

**Key messages:**

• Digital-based intervention would be an effective way for vulnerable ethnic minority adolescents to engage in healthy diet.

• The Living Lab approach was used as an essential strategy to develop a digital health intervention to improve the healthy diet of ethnic minority adolescents.

